# The Relief and Cure of Spinal Curvatures

**Published:** 1898-06

**Authors:** 


**The Relief and Cure of Spinal Curvatures.** By Dr. Percy G. Lewis. Pp. xii., 208. London: John Bale, Sons & Danielsson, Ltd. 1897.

There is nothing very original in this small book, which is written "for the General Practitioner by a General Practitioner." It is illustrated by a large number of photographs of cases which have been under the author's care, but most of them are of little value in conveying information to the reader. The chapters on treatment are eminently practical and judicious. Mechanical treatment is advised to a slight extent only, and reliance is placed chiefly on muscular exercises, a very good description of which is appended. The author quotes largely from Roth, Barwell, Brodhurst, Noble Smith and other writers; and if he does not give us anything new of his own, he at least recommends the best work of others. The book may be looked upon as a trustworthy guide in the treatment of spinal curvatures.

